# Determination of 13-*cis*-Retinoic Acid and Its Metabolites in Plasma by Micellar Electrokinetic Chromatography Using Cyclodextrin-Assisted Sweeping for Sample Preconcentration

**DOI:** 10.3390/molecules26195865

**Published:** 2021-09-28

**Authors:** Ying-Xuan Huang, Yu-Ying Chao, Yi-Hui Lin, Jing-Ru Liou, Hai-Chi Chan, Yen-Ling Chen

**Affiliations:** 1 Department of Fragrance and Cosmetic Science, College of Pharmacy, Kaohsiung Medical University, Kaohsiung 807, Taiwan; uivi95251@gmail.com; 2 Department of Public Health, College of Health Sciences, Kaohsiung Medical University, Kaohsiung 807, Taiwan; yuyich@kmu.edu.tw; 3 School of Pharmacy, College of Pharmacy, China Medical University, Taichung 406, Taiwan; yihulin@mail.cmu.edu.tw; 4 Department of Pharmacy, Kaohsiung Medical University Hospital, Kaohsiung Medical University, Kaohsiung 807, Taiwan; vvv1050002@gmail.com; 5 Department of Chemistry and Biochemistry, National Chung Cheng University, Chia-Yi 621, Taiwan; x450c93fu@gmail.com; 6 Center for Nano Bio-Detection, National Chung Cheng University, Chia-Yi 621, Taiwan

**Keywords:** 13-*cis*-retinoic acid, metabolites, cyclodextrin-assisted sweeping, protein precipitation, liquid–liquid extraction, plasma

## Abstract

The online preconcentration technique, cyclodextrin-assisted sweeping (CD-sweeping), coupled with micellar electrokinetic chromatography (MEKC) was established to determine 13-cis-retinoic acid (13-*cis*-RA), all-*trans*-retinoic acid (all-*trans*-RA) and 4-oxo-13-*cis*-retinoic acid (4-oxo-13-*cis*-RA) in human plasma. A CD-sweeping buffer (45 mM borate (pH 9.2), containing 80 mM sodium dodecyl sulfate (SDS) and 22 mM hydroxypropyl β-CD (HP-β-CD) was introduced into the capillary and, then, the sample dissolved in 70 mM borate (pH 9.2): methanol = 9:1 (*v/v*) was injected into capillary by pressure. The separation voltage was 23 kV. Compared to the conventional cyclodextrin-micellar electrokinetic chromatography (CD-MEKC) method, the new technique achieved 224–257-fold sensitivity enrichment of analytes. The limits of detection of 13-*cis*-RA, all-*trans*-RA were 1 ng/mL, whereas that of 4-oxo-13-*cis*-RA was 25 ng/mL in plasma. The linear ranges of 13-*cis*-RA, all-*trans*-RA were between 15 and 1000 ng/mL, whereas that of 4-oxo-13-*cis*-RA was between 75 and 1500 ng/mL. The coefficient of correlation between the concentration of analytes and peak area ratio of analytes and internal standard (2, 4-dihydroxy-benzophenone) for intra-day (*n* = 3) and inter-day (*n* = 5) analyses were both greater than 0.999. The optimized experimental conditions were successfully applied to determine 13-*cis*-retinoic acid and its metabolites in plasma samples from a patient during the administration of 13-*cis*-RA for treating acne.

## 1. Introduction

13-*cis*-retinoic acid (13-*cis*-RA), an anti-acne drug, is also known as isotretinoin. It can reduce the activity of 5α-reductase, decrease conversion of testosterone to active dihydrotestosterone (DHT) and inhibit sebum secretion. In addition, it can inhibit the proliferation of *Propionibacterium acnes* [1]. 13-*cis*-RA exhibits a high proportion of protein binding (>99.9%) in plasma. According to the metabolic pathway of 13-*cis*-RA (Figure S1), it was metabolized by liver enzymes (CYP2C8, CYP2C9, CYP3A4 and CYP2B6) and converted to all-*trans*-retinoic acid (all-*trans*-RA), 4-oxo-13-*cis*-retinoic acid (4-oxo-13-*cis*-RA) and 4-oxo-all-*trans*-retinoic acid (4-oxo-all-*trans*-RA). Approximately 20%–30% of 13-*cis*-RA can convert to all-*trans*-RA [2,3]. 4-oxo-13-*cis*-RA and 4-oxo-all-*trans*-RA are seldom converted between each other in plasma [2,4]. All-*trans*-RA can bind with the retinoic acid receptor on epidermal cells to stimulate the differentiation of keratinocytes, decrease the cohesion between keratinocytes and then make keratinocytes normalize [5]. The adverse effect of 13-*cis*-RA was dry mouth and skin, dry eye, back pain, arthralgia, epistaxis, headache, nasopharyngitis, dermatitis and teratogenicity [6,7]. In order to evaluate the pharmacokinetics of 13-*cis*-RA, 13-*cis*-RA and its major metabolites, all-*trans*-RA and 4-oxo-13-*cis*-RA were chosen to test in plasma sample. 

**Figure 1:**
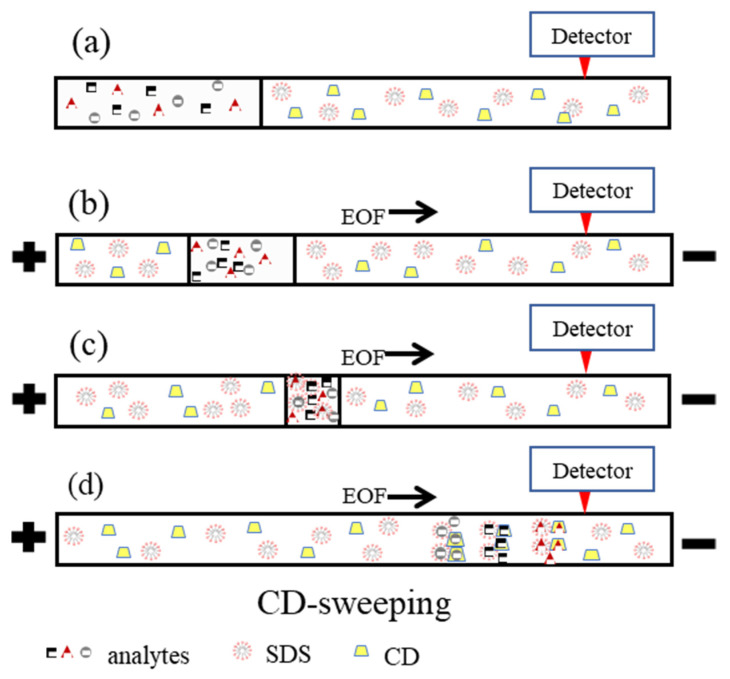
The mechanism of CD-sweeping. (**a**) sample injection (**b**) voltage applied. (**c**) sweeping (**d**) CD-MEKC separation.

Capillary electrophoresis (CE) is widely used in drug concentration monitoring [8] and pharmaceutical analysis [9,10,11]. It has the advantages such as high separation efficiency, low sample and solvent consumption. However, the concentration sensitivity of UV-absorption detection in CE is usually insufficient in quantifying drugs in plasma or urine. In this study, the CE combined with online concentration technique was established to improve the detection sensitivity of 13-*cis*-RA and its metabolites in the plasma sample. A schematic of cyclodextrin-assisted sweeping (CD-sweeping) for sample preconcentration is shown in Figure 1. First, a sweeping buffer, containing sodium dodecyl sulfate (SDS) micelles and CD with high pH value was directed into a capillary. Then, a large sample plug was injected into the capillary (Figure 1a). After applying positive voltage, the analytes with negative charges migrate to the boundary of the sample zone and sweeping buffer and stack at the boundary (Figure 1b). Moreover, the electrophoretic mobility of sodium dodecyl sulfate (SDS) micelles is larger than the anionic analytes and analytes were swept using SDS micelles from the outlet to the inlet of the capillary (Figure 1c). Finally, analytes migrate to the detection by cyclodextrin-micellar electrokinetic chromatography (CD-MEKC) mode (Figure 1d) [12,13,14,15]. The present study aims to establish an efficient analytical method for determining 13-*cis*-RA and its major metabolites, all-*trans*-RA and 4-oxo-13-*cis*-RA in plasma by CD-sweeping for sample preconcentration in capillary.

## 2. Results and Discussion

### 2.1. Optimization of the Protein Precipitation with Liquid-Liquid Extraction (PP-LLE) Conditions

Light and oxygen degrade 13-*cis*-RA and all-*trans*-RA due to the presence of conjugated double bonds. The isomers can be interconverted in plasma when thiol-containing compounds, such as glutathione, mercaptoethanol and apoferritin, exist in plasma [16]. To maintain the stability of RA, the antioxidant of l-ascorbic acid and thiol-blocking reagent of *N*-ethylmaleimide (NEM) was added to the plasma [17]. The plasma protein will denature and precipitate in the capillary when interacting with organic solvent [18]. Therefore, separating the protein from the plasma sample is important for avoiding capillary blockage before the samples are injected into CE. Compared with other extraction methods, such as solid-phase extraction, PP-LLE was chosen as the sample pretreatment method because of its low reagent consumption, low cost and ease of operation. Several parameters of PP-LLE were evaluated to obtain the optimized condition, including the pH value of plasma, types and volume of the protein precipitation reagents, as well as the types and volume of the extracting solvent. 

#### 2.1.1. Effects of pH Value of Plasma

The pKa values of acidic 13-*cis*-RA and its metabolites are about 4.76; most analytes appear protonated under low pH conditions [19]. In our study, the extraction efficiency was evaluated by adjusting different pH values of plasma (pH 2.0, 3.0 and 7.4). The results show that none of the peaks were observed when the pH value of plasma was in a neutral condition. Most analytes were deprotonated and not easily partitioned into the extraction solvent in plasma having pH 7.4. When the pH value of plasma was adjusted to 2.0 and 3.0, most analytes appeared protonated and partitioned into extraction solvent. Three peaks can be observed at pH 2.0 and 3.0, respectively. All analytes have a similar extraction efficiency at pH 2.0 and 3.0. The results are shown in Figure S2. The relative concentration was the ratio of the peak area of analytes to the peak area of internal standard (I.S.)(2, 4-dihydroxy-benzophenone, BP1) and the maximum was set as 100%. Finally, the pH value of plasma was adjusted to 3.0 and selected as the optimal condition.

#### 2.1.2. Effects of Protein Precipitation Reagents 

**Figure 2:**
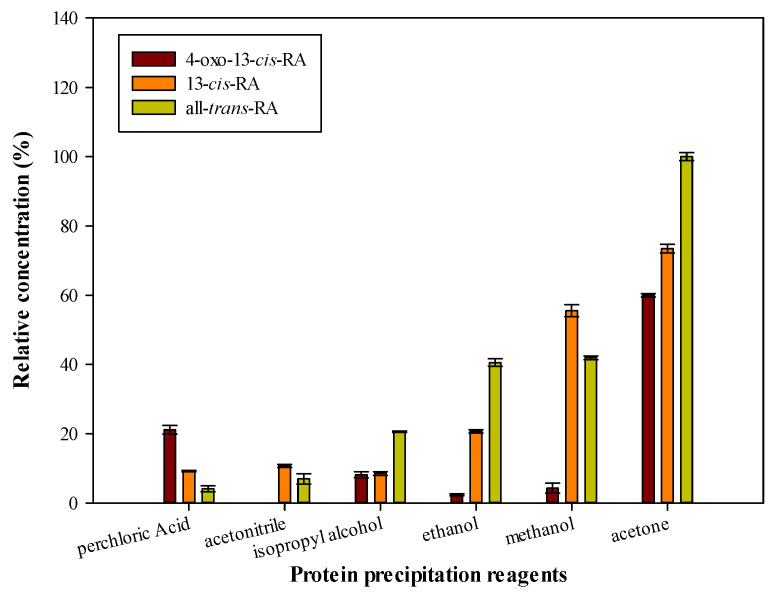
Effects of different protein precipitation reagents in PP-LLE procedure. Analyte concentrations: 4-oxo-13-*cis*-RA: 600 ng/mL, 13-*cis*-RA: 300 ng/mL, all-*trans*-RA: 300 ng/mL.

Strong acid and organic solvent were used as protein precipitation reagents in plasma sample pretreatment because they can disrupt the secondary and tertiary structures of protein [20]. The proteins can form insoluble aggregates and precipitation when strong acid such as trichloroacetic and metaphosphoric acids are added to plasma [18]. However, most analytes remained unstable under extremely acidic conditions. Organic solvents, such as acetonitrile, acetone, methanol and ethanol can decrease the relative permittivity of plasma and protein solubility. Two types of protein precipitation reagents (strong acids and organic solvents) were chosen to evaluate the extraction efficacy, and the results are shown in Figure 2. The volume of perchloric acid was set at 30 μL, acetonitrile, Isopropyl alcohol and ethanol were set at 1000 μL, respectively. Among them, acetone as a precipitation reagent can obtain better extraction efficiency.

**Figure 3:**
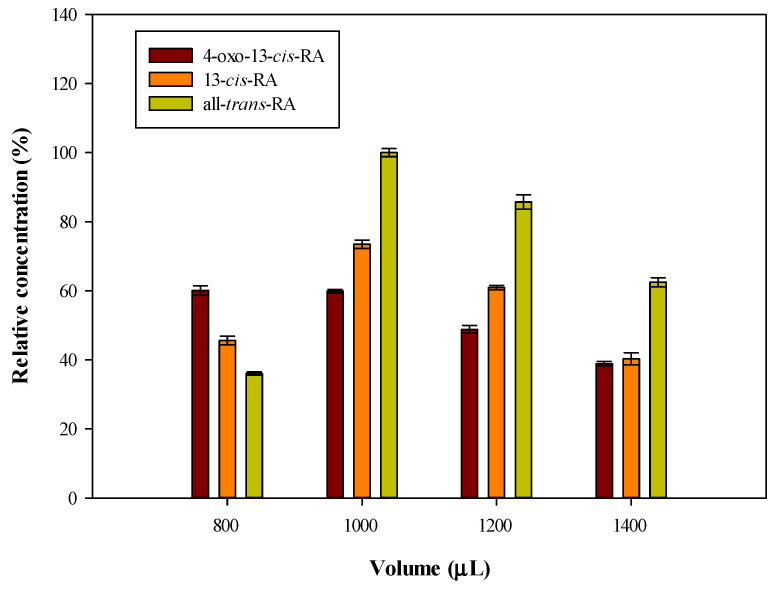
Effects of different volume of acetone in PP-LLE procedure. Analyte concentrations: 4-oxo-13-*cis*-RA: 600 ng/mL, 13-*cis*-RA: 300 ng/mL, all-*trans*-RA: 300 ng/mL.

According to Blanchard et al., about 99.4% of plasma protein were precipitated when the volume ratio of acetone and plasma was set to 2:1 [21]. To evaluate extraction efficiency, different volumes of acetone as a protein precipitation reagent (800, 1000, 1200 and 1400 µL) were investigated. The results are shown in Figure 3. When adding 1000 µL acetone to 500-µL plasma, the relative response was 100%, 76% and 60% for all-*trans*-RA, 13-*cis*-RA and 4-oxo-13-*cis*-RA, respectively. In addition, excessive acetone in the solution will decrease the extraction efficiency because the increasing proportion of the acetone increases the polarity of the extraction solvent. Finally, acetone was chosen as the precipitation reagent and the volume was set to 1000 µL.

#### 2.1.3. Effects of Extracting Solvent

According to the structures of 13-*cis*-RA and its metabolites, three extracting solvents (hexane, toluene and chloroform) that were immiscible or slightly miscible with acetone were chosen to evaluate the extraction efficacy. Only 4-oxo-13-*cis*-RA with a lower log P (4.53) value than 13-*cis*-RA (5.66) and all-*trans*-RA (6.3) can be observed when extracted using chloroform [19,22]. All analytes were extracted using hexane and toluene. The higher extraction efficiency was acquired using hexane extraction because of the long hydrocarbon chains of RA. Volumes of 100, 150, 200, 250 and 300 µL hexane were chosen to investigate their extraction effect. We observed that the extraction efficiency increased as hexane volume increases to 250 µL. When the volume of hexane increased to 300 µL, a decreasing peak signal was observed. Therefore, 250 µL of hexane was selected as the optimal volume of hexane.

#### 2.1.4. Compare the Different Extraction Methods

To investigate the necessity of protein precipitation, three kinds of extraction methods, including only protein precipitation (PP), only liquid–liquid extraction (LLE) and PP combined with LLE were designed to explore the extraction efficiency of 13-*cis*-RA and its metabolites in plasma. The procedure of protein precipitation was as fellow: the pH values of plasma samples were adjusted to 3 and then adding 1000 µL acetone to precipitate protein. All analytes can be extracted using only LLE and PP-LLE methods, however, no peaks were observed when using only PP as the extraction method. It cannot effectively remove matrix interference. Compared with LLE, the extraction efficiency of 13-*cis*-RA and all-*trans*-RA can increase 4- and 10-fold using PP-LLE, respectively.

According to the above discussion, the optimized PP-LLE conditions were as follows: The pH value of plasma was adjusted to 3.0 using hydrochloric acid; then, 1000 µL acetone was added as a protein precipitation reagent. After vortexing and centrifugation at 10,625× *g* for 10 min, the supernatant was extracted using hexane (250 µL). 

### 2.2. Optimization of CD-Sweeping Conditions

To optimize the sensitivity and separation efficiency of the CD-sweeping method, the conditions of sweeping buffer, the sample solution and injection amount were investigated. Because 13-*cis*-RA and all-*trans*-RA were unstable in the acidic condition [8], three basic buffers including borate, Tris and glycine were investigated. All analytes can be observed in borate and glycine buffer. In addition, the high ionic strength of borate buffer can decrease the EOF, prolong the separation time and enhance the sweeping ability. Therefore, the borate buffer was used in this study.

#### 2.2.1. SDS Concentration in the Sweeping Buffer

Sweeping, a powerful online sample concentration technique, is developed to improve the sensitivity of CE. The anionic micelles were used as a pseudostationary phase in the separation buffer to interact with analytes, thus, accumulating analytes in the capillary [23,24]. Four SDS concentrations (60, 80, 100 and 120 mM) in the sweeping buffer were chosen to evaluate the sweeping ability. The results indicated that 13-*cis*-RA and its metabolites can be separated in these conditions. When increasing SDS concentrations, more analytes are distributed to the micelle and prolong the total analysis time. Considering the analysis time and peak intensity, 80 mM SDS was selected as the optimal concentration.

#### 2.2.2. HP-β-CD Concentrations in Sweeping Buffer

13-*cis*-RA can convert to all-*trans*-RA isomers. To improve the sweeping ability and isomer resolution, organic solvents or cyclodextrins (CDs) were used as separation buffer additives [25,26,27,28]. Adding organic solvent in the sweeping buffer can reduce the polarity of buffer, alter the distribution of the analyte between micellar and buffer and decrease EOF. In the preliminary experiment, 13-*cis*-RA and all-*trans*-RA could be separated during the addition of different types of organic solvents (methanol, ethanol and acetonitrile). However, the poor stacking effect of 4-oxo-13-*cis*-RA was found. The architecture of CDs includes a hydrophilic part on the molecular exteriors and a hydrophobic cavity on the molecular interiors. Cyclodextrins can be divided into α-CD, β-CD and γ-CD comprising six, seven and eight oligosaccharide monomers, respectively. The inner cavity of CD is hydrophobic; thus, the hydrophobic analytes can distribute to the inner cavity and form a complex through hydrogen bond or dipole-induced dipole interaction [29]. The analytes can distribute to the inner cavity of CD with different proportions due to the geometry difference of isomers, which can be used to increase the CE-separation efficiency [30].

**Figure 4:**
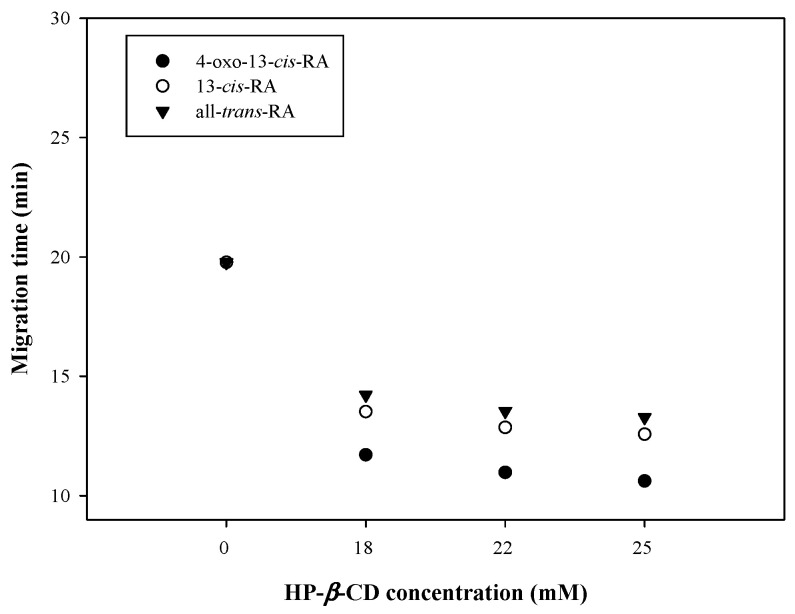
Effects of different HP-β-CD concentrations on migration time of analytes.

The results showed that β-CD can improve the separation efficiency by altering the effective electrophoretic mobility of analytes. Because of low solubility of β-CD, three derivates of β-CD with higher solubility (sulfated β-CD, heptakis (2, 3, 6-tri-*o*-methyl) β-CD (heptakis-β-CD) and hydroxypropyl β-CD (HP-β-CD)) were used to evaluate the separation efficiency. Three peaks were observed when HP-β-CD was selected; analytes could not be separated without adding HP-β-CD and the result is shown in Figure 4. All analytes could be determined within 15 min when the concentration of HP-β-CD was more than 18 mM. The polyene chain structure of 13-*cis*-RA and all-*trans*-RA can enter the hydrophobic cavity of HP-β-CD and form a complex [31]. Thus, the difference in stereochemical structures of 13-*cis*-RA and all-*trans*-RA causes the different distribution ratios in HP-β-CD and enables separation of the isomers. The symmetry of the peak can be calculated using the asymmetry factor (As). The equation is As = B/A. A vertical line from the highest point of the wave peak and a horizontal line under 10% of the wave crest height, intersect at one point. The point to the front end of the horizontal line can be defined as A, whereas the back one can be regarded as B [32]. When the value of As is close to 1, the peak shows more symmetry; if As > 1, the peak is tailing; if As < 1, the peak is fronting. The 22 mM HP-β-CD in sweeping buffer possesses better peak symmetry, thus, it was selected as the optimal condition (Figure S3).

#### 2.2.3. Borate Buffer Concentrations in the Sample Solution

The electrophoretic mobility of analytes and the sweeping ability of micelle could be affected by the borate buffer concentration in the sample solution. Four concentrations of borate buffer solution (30, 50, 70 and 90 mM) were investigated. In addition, the signal intensity of analytes was improved with an increase in the concentration of borate buffer solution. This implied that low electrophoretic mobility of analytes under high borate buffer concentrations could enhance the sweeping ability of SDS. Moreover, the conductivities of four borate buffer concentrations in the sample solution were 3.22, 4.98, 6.37 and 8.01 mS/cm, respectively. The conductivity of the 45 mM borate-sweeping buffer was 8.14 mS/cm. The conductivity of the sample solution and sweeping buffer solution is slightly different in the 90 mM borate buffer; therefore, the field-enhance effect was reduced (data are shown in Figure S4). Finally, 70 mM borate buffer had the highest intensity and it was chosen as the optimal condition.

#### 2.2.4. Methanol Proportion in the Sample Solution

13-*cis*-RA and its metabolites are hydrophobic compounds and methanol was added to the sample solution to increase the solubility of analytes and improve the stacking efficiency. Different proportions of methanol (5%, 10%, 15% and 20%) in the sample solution were tested in this study. All analytes acquired a good resolution when methanol is added to the sample solution. The highest relative response was obtained when 10% methanol was added because the analytes were dissolved in the sample solution and increase the sweeping ability. However, excess methanol amount would reduce the sweeping ability of SDS with low sensitivity. The 10% methanol addition, possessing a higher peak response, was selected as the optimal separation condition.

#### 2.2.5. The Injection Sample Amount

Four sample injection pressures (13.8, 27.6, 41.4 and 55.1 kPa) were studied to evaluate the stacking efficiency. The injection lengths (the ratio of the injection length to the total capillary length) were 8.1 (13.5%), 16.1 (26.7%), 24.2 (40.2%) and 32.2 (53.5%) cm in the capillary, respectively. The signal intensity of the analytes was increased with a larger amount of sample injection. However, with the larger sample injection pressure, the length of analyte separation was shorter, which can affect the resolution of analytes and interference peak. When the injection pressure achieved 55.1 kPa, the interference peak was overlapped with all-*trans*-RA. Therefore, the injection pressure at 41.4 kPa was considered the optimal condition in this study.

### 2.3. Selective Experiment

**Figure 5:**
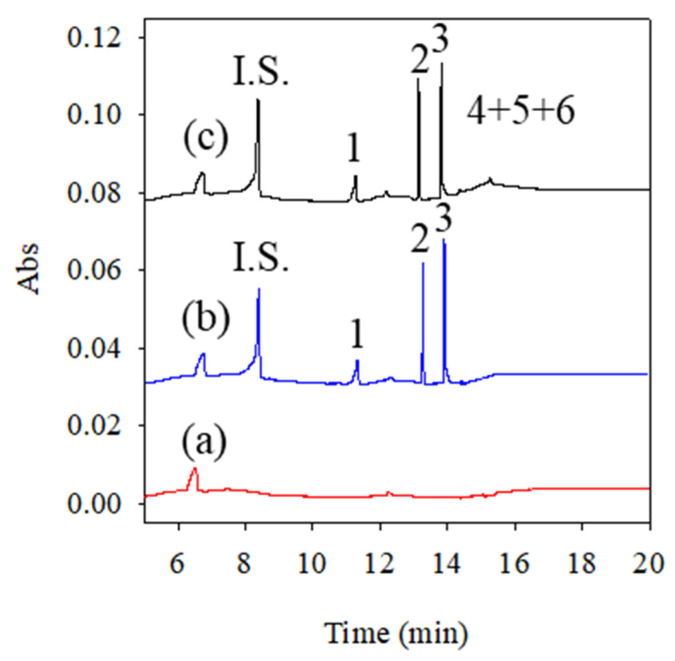
Electropherograms of selective experiment on 13-*cis*-RA, all-*trans*-RA, 4-oxo-13-*cis*-RA, all-*trans*-retinol, all-*trans*-retinal, retinyl acetate in plasma sample. (**a**) blank, (**b**) 13-*cis*-RA, all-*trans*-RA, 4-oxo-13-*cis*-RA, (**c**) 13-*cis*-RA, all-*trans*-RA, 4-oxo-13-*cis*-RA, all-*trans*-retinol, all-*trans*-retinal, retinyl acetate. Analyte concentrations: I.S.: BP-1 (2 µg/mL), peak 1: 4-oxo-13-*cis*-RA (600 ng/mL), peak 2: 13-*cis*-RA (300 ng/mL), peak 3: all-*trans*-RA (300 ng/mL), peak 4: all-*trans*-retinol (10 µg/mL), peak 5: all-*trans*-retinal (10 µg/mL), peak 6: retinyl acetate (10 µg/mL).

β-Carotene was metabolized to endogenous retinoid compounds, such as all-*trans*-retinol, all-*trans*-retinal and retinyl acetate. To confirm these, endogenous retinoid compounds will not interfere with the analysis of 13-*cis*-RA and its metabolites in plasma. Endogenous retinoid compounds were spiked in plasma before PP-LLE and then analyzed using CD-sweeping. In Figure 5, 4-oxo-13-*cis*-RA is observed, followed by 13-*cis*-RA, all-*trans*-RA and three retinoid compounds, which include all-*trans*-retinol, all-*trans*-retinal and retinyl acetate. The migration time of all-*trans*-retinol, all-*trans*-retinal and retinyl acetate was within 15–18 min. Endogenous retinoid compounds exhibited poor resolution in this analysis method and showed non-overlapped peaks with 13-*cis*-RA and its metabolites. It demonstrated that the established analytical method, in this study, showed good selectivity.

### 2.4. Analytical Figures of Merit 

The limits of detection (LOD) defined as the ratio of the signal-to-noise (S/N) is 3:1. The LOD values of 13-*cis*-RA, all-*trans*-RA and 4-oxo-13-*cis*-RA were 1, 1 and 25 ng/mL, respectively. The linear ranges of 13-*cis*-RA, all-*trans*-RA and 4-oxo-13-*cis*-RA were set to 15–1000 ng/mL, 15–1000 ng/mL and 75–1500 ng/mL, respectively. In the regression equation, x was defined as the concentration of analytes and y was the peak area ratio of analytes and internal standard (BP-1). Investigation of linear relationships was performed using the least-square method. The coefficient of correlation (γ) between the concentration of analytes and peak area ratio of analytes and internal standard for intra-day (*n* = 3) and inter-day (*n* = 5) analyses were both greater than 0.999. We selected three points at low, medium and high concentration in the linear range of the calibration curve for evaluating the precision and accuracy of this method. Accordingly, the concentrations of 13-*cis*-RA, all-*trans*-RA were 30, 250 and 950 ng/mL, respectively and 4-oxo-13-*cis*-RA were 100, 400 and 1400 ng/mL, respectively. In the intra-day analysis, the relative standard deviation (RSD) values of the three analytes were less than 4.34% and the relative error (RE) values were less than 5.71%. In the inter-day analysis, the RSD values were less than 4.18% and the RE values were less than 6.13% (data are shown in Table 1). This result indicated that the established method has good precision and accuracy.

### 2.5. CD-Sweeping Sensitivity Enhancement

A conventional CD-MEKC was compared with CD-sweeping to evaluate the sensitivity enhancement. The separation conditions for the CD-MEKC method were set to a pressure of 3.5 kPa for 5 s for sample injection; the sample solution was 70 mM borate: methanol = 9:1 (*v/v*) and the separation condition, was 45 mM borate buffer, which contained 80 mM SDS and 22 mM HP-β-CD. Compared with CD-MEKC, a larger amount of sample (41.4 kPa for 40 s) was injected into capillary for CD-sweeping method. The sensitivity enhancement equation is shown in Equation (1). The sensitivity enhancement for 13-*cis*-RA, all-*trans*-RA and 4-oxo-13-*cis*-RA were 257-, 224- and 255-fold, respectively.

(1) $$\mathrm{Sensitivity}\;\mathrm{enhancement}\;=\frac{\mathrm{Peak}\;\mathrm{area}\;\left(\mathrm{CD}-\mathrm{sweeping}\right)}{\mathrm{Peak}\;\mathrm{area}\;\left(\mathrm{CD}-\mathrm{MEKC}\right)}\times \frac{C\;\left(\mathrm{CD}-\mathrm{MEKC}\right)}{C\;\left(\mathrm{CD}-\mathrm{sweeping}\right)\;}$$

C (CD-MEKC): the concentration of analytes using CD-MEKC

C (CD-sweeping): the concentration of analytes using CD-sweeping

### 2.6. Application

**Figure 6:**
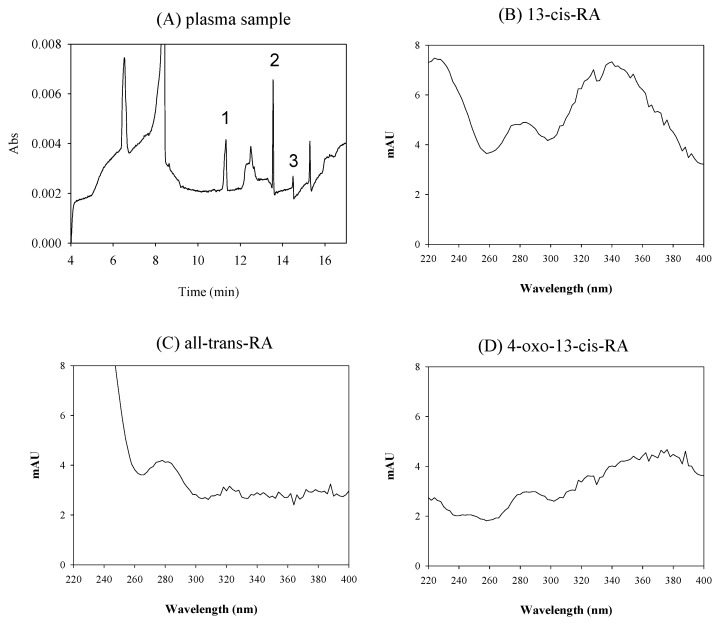
Electropherograms of (**A**) plasma sample from patient in the treatment of acne by 13-*cis*-RA in PP-LLE coupled with CD-sweeping. Peak 1: 4-oxo-13-*cis*-RA, peak 2: 13-*cis*-RA, peak 3: all-*trans*-RA. UV spectra of (**B**) 13-*cis*-RA, (**C**) all-*trans*-RA and (**D**) 4-oxo-13-*cis*-RA in plasma sample.

To treat acne, a female (23 years old, 54 kg) continues taking Roaccutane^®^ (13-*cis*-RA, 10 mg) once daily. The human plasma was collected 2 h after taking Roaccutane^®^ and was analyzed using CD-sweeping in Figure 6. The concentrations of 13-*cis*-RA and 4-oxo-13-*cis*-RA in the plasma sample were 40.35 ± 0.32 ng/mL and 175.47 ± 12.11 ng/mL, respectively. According to pharmacokinetic studies, the concentration of 4-oxo-13-*cis*-RA was 2 to 5-fold that of 13-*cis*-RA [10]. The concentration of all-*trans*-RA was lower than LOQ in this sample. In this study, the analyte compositions were confirmed using UV spectra of 4-oxo-13-*cis*-RA and 13-*cis*-RA. It can be observed the wavelength of maximum absorbance of 4-oxo-13-*cis*-RA and 13-*cis*-RA were 365 nm and 350 nm, respectively, from the UV spectra. However, the concentration of all-*trans*-RA was lower than LOQ, we cannot obviously observe its wavelength of maximum absorbance at 350 nm. According to the experimental data, it can prove that this method is suitable for the determination of 13-*cis*-RA and its metabolites in plasma samples.

## 3. Materials and Methods

### 3.1. Reagents and Chemicals

The chemicals and reagents used were of analytical grade. 13-*cis*-RA, all-*trans*-RA, 4-oxo-13-*cis*-RA, all-*trans*-retinol, all-*trans*-retinal, retinol acetate, l-ascorbic acid, *N*-ethylmaleimide (NEM), 2, 4-dihydroxy-benzophenone (BP-1), ethanol, (sulfate sodium salt)-β-cyclodextrin (Sulfate-β-CD) and heptakis (2,3,6-tri-*o*-methyl)-β-cyclodextrin (Heptakis-β-CD) were purchased from Sigma-Aldrich (St. Louis, MO, USA). Isopropyl alcohol, disodium tetraborate decahydrate (borate), glycine, tris (hydroxy-methyl)aminomethane (tris), sodium dodecyl sulfate (SDS), hydrochloric acid (HCl) and sodium hydroxide (NaOH) were purchased from Merck (Darmstadt, Germany). Methanol and acetone were purchased from Macron Fine Chemicals (Center Valley, PA, USA). Acetonitrile was purchased from J. T. Baker (Phillipsburg, NJ, USA). Perchloric acid and hexane were purchased from Honeywell (Seelze, Germany). Ultrapure water was obtained from the Milli-Q^®^ system and was used to prepare the experimental solution. α-cyclodextrin (α-CD), β-cyclodextrin (β-CD), γ-cyclodextrin (γ-CD), Hydroxypropyl-β-cyclodextrin (HP-β-CD) were purchased from Tokyo Chemical Industry CO., LTD (Tokyo, Japan). The standard solutions (13-*cis*-RA, all-*trans*-RA and 4-oxo-13-*cis*-RA) and internal standard (BP-1) were dissolved in methanol and stored at −20 °C.

### 3.2. CE Conditions

All CE experiments were conducted using the MDQ P/ACE system with a photodiode array (PDA) detector (Beckman Coulter Inc., Fullerton, CA, USA). The uncoated fused-silica capillaries (50 µm I.D and 365 nm O.D) were purchased from Polymicro Technologies (Phoenix, AZ, USA). The effective length of the capillary was 50 cm to the detection window and the total length of the capillary was 60.2 cm. The detection wavelength was set to 350 nm. For the CD-sweeping method, the sample solution consisted of 70 mM borate (pH 9.2): methanol = 9:1 (*v/v*). The sweeping buffer was 45 mM borate (pH 9.2), containing 80 mM SDS and 22 mM HP-β-CD. The capillary was rinsed using ultrapure water (3 min), between consecutive analyses, followed by 1 M NaOH (3 min), ultrapure water (3 min) and rinsing buffer (3 min). The separation voltage was set to 23 kV and the electric current was 92 μA. The sample injection pressure was set at 41.4 kPa for 40 s and the injection length was 24.2 cm. For the CD-MEKC method, the sample solution and sweeping buffer were the same as the CD-sweeping method. The sample injection pressure was set at 3.5 kPa for 5 s (2.5 cm injection length) in the conventional CD-MEKC method. 

### 3.3. Plasma Sample Pretreatment

The extraction procedure of PP-LLE was as follows: 500 µL human plasma sample with 5 µL NEM (4.8 mM) and 5 µL L-ascorbic acid (14.8 mM) was adjusted to pH 3.0 using 1 M HCl. The mixture was mixed with 1000 µL acetone by vortexing for 1 min and centrifuged at 10,625× *g* for 10 min. The supernatant layer was transferred to another microcentrifuge tube and mixed with 250 µL hexane by vortexing for 1 min and centrifuged at 12,000 rpm for 10 min. Then, the supernatant layer was collected and evaporated until dry. The residue sample was reconstituted with sample solution before the injection of the CE experiment.

## 4. Conclusions

In this study, 13-*cis*-RA, all-*trans*-RA and 4-oxo-13-*cis*-RA were extracted from plasma using the PP-LLE procedure and separated using the CD-sweeping online concentration technique within 15 min. The CD-sweeping could improve sensitivity up to 224–257 times the CD-MEKC method. The LOD of 13-*cis*-RA and all-*trans*-RA were 1 ng/mL, whereas the LOD of 4-oxo-13-*cis*-RA was 25 ng/mL in plasma. The developed method can further be applied to acne patients who took the 13-*cis*-RA.

## Figures and Tables

**Table 1:** Precision and accuracy analysis for intra-day (*n* = 3) and inter-day (*n* = 5) analysis of 13-*cis*-RA and its metabolites.

Concentration (ng/mL)	Intra-Day (*n* = 3)		Inter-Day (*n* = 5)	
	RSD (%)	RE (%)	RSD (%)	RE (%)
13-*cis*-RA				
30	2.56	−0.65	3.07	0.84
250	3.50	−1.71	2.93	−0.58
950	2.19	−0.97	3.52	1.28
All-*trans*-RA				
30	1.74	−0.65	1.17	−1.87
250	4.34	−0.65	4.18	0.87
950	0.93	0.48	2.47	−1.25
4-oxo-13-*cis*-RA				
100	1.21	0.93	1.75	−0.36
400	0.94	5.71	1.31	6.13
1400	1.73	−1.53	3.66	0.80

RE: (concentration found − concentration known)/(concentration known)*100.

## Data Availability

Not applicable.

## Supplementary Materials

The following are available online, Figure S1: Metabolic pathway of the 13-*cis*-RA. Figure S2: Effects of different plasma pH values in PPT-LLE procedure. Analyte concentrations: 4-oxo- 13-*cis*-RA: 600 ng/mL, 13-*cis*-RA: 300 ng/mL, all-*trans*-RA: 300 ng/mL. Figure S3: Effects of different concentrations of HP-β-CD on asymmetry factor of analytes. Figure S4: Effects of different concentrations of borate buffer in sample solution. Analyte concentrations: 4-oxo-13-*cis*-RA: 600 ng/mL, 13-*cis*-RA: 300 ng/mL, all-*trans*-RA: 300 ng/mL.

## Sample Availability

Samples of the compounds are not available from the authors.
