# Supplementary material for: Determination of 13-cis-Retinoic Acid and Its Metabolites in Plasma by Micellar Electrokinetic Chromatography Using Cyclodextrin-Assisted Sweeping for Sample Preconcentration
Source: Molecules. 2021 Sep 28;26(19):5865. doi: 10.3390/molecules26195865 (PMC8512417; doi:10.3390/molecules26195865)
Supplement: Supplementary file 1 [file molecules-26-05865-s001.zip › molecules-1370988-supplementary.pdf]

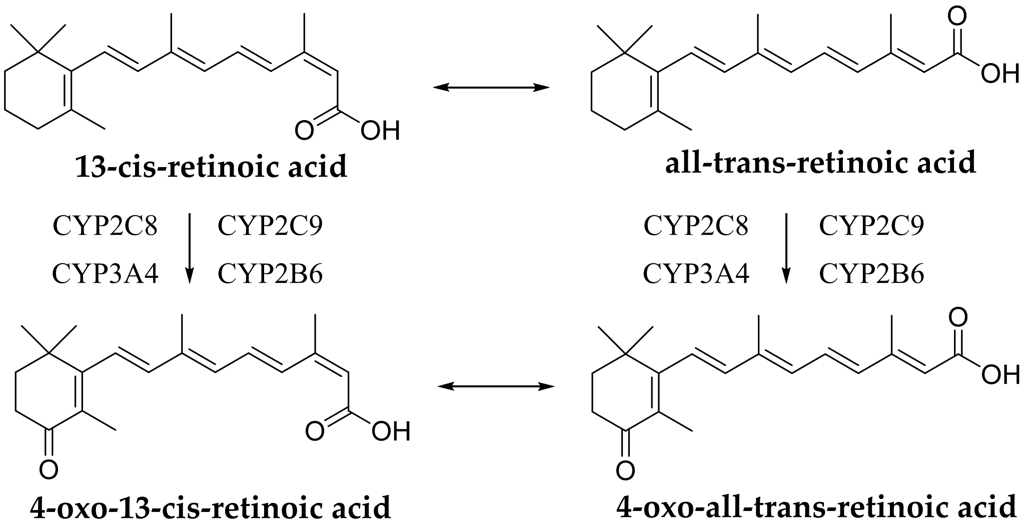

Figure S1. Metabolic pathway of the 13-cis-RA.

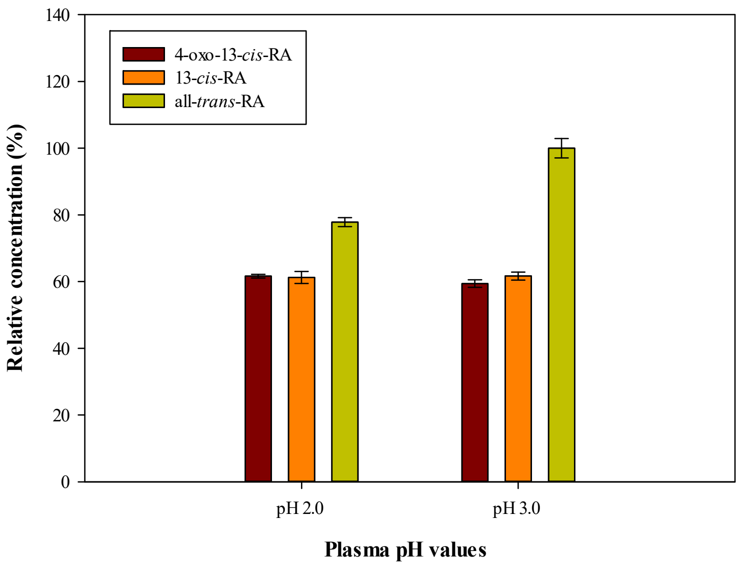

Figure S2. Effects of different plasma pH values in PPT-LLE procedure. Analyte concentrations: 4-oxo- 13-cis-RA: 600 ng/mL, 13-cis-RA: 300 ng/mL, all-trans-RA: 300 ng/mL.

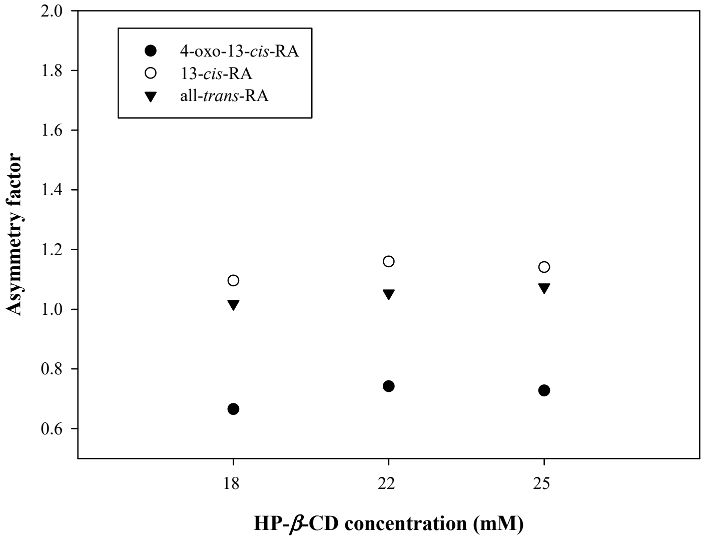

Figure S3. Effects of different concentrations of HP-β-CD on asymmetry factor of analytes.

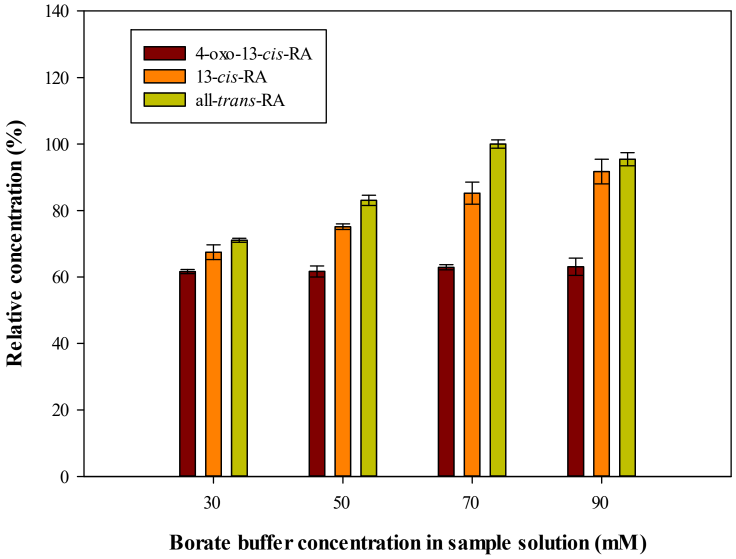

Figure S4. Effects of different concentrations of borate buffer in sample solution. Analyte concentrations: 4-oxo-13-cis-RA: 600 ng/mL, 13-cis-RA: 300 ng/mL, all-trans-RA: 300 ng/mL. .
